# Acute Thrombocytopaenia Induced by Teicoplanin in a Patient With Right Leg Cellulitis

**DOI:** 10.7759/cureus.93653

**Published:** 2025-10-01

**Authors:** Muhammad Ansar, Daneer S Rahanu, Nyein Su Wai, Wutyee Phoo, Md Nahid Hasan, Abdul Nabi Saleemi

**Affiliations:** 1 Internal Medicine, University Hospitals Plymouth NHS Trust, Plymouth, GBR; 2 General Internal Medicine, University Hospitals Plymouth NHS Trust, Plymouth, GBR; 3 Cardiology/Acute Medicine, University Hospitals Plymouth NHS Trust, Plymouth, GBR

**Keywords:** antibiotics side effect, drug-induced thrombocytopaenia, low platelet count, thrombocytopaenia, wound infections

## Abstract

Platelets are one of the important cells in the human blood that regulate the coagulation system. Abnormalities in platelets may lead to thrombosis or bleeding. Acute thrombocytopaenia carries a wide differential in a hospitalised patient, and drug-induced thrombocytopaenia is one of the uncommon findings. This case report describes teicoplanin-induced thrombocytopaenia in a patient who was being treated with this antibiotic and then developed thrombocytopaenia to a critically low level. This low level of platelets predisposes to bleeding, either minor or major. However, prompt recognition and stopping the offending drug reverse the process of ongoing thrombocytopaenia.

## Introduction

Thrombocytopaenia is defined as a platelet count of less than 150,000/microL. This is a common finding in a full blood count report for patients hospitalised for various reasons. This is mostly due to infections and sepsis and could be drug-induced as well. Drug-induced thrombocytopaenia is associated with a drop in platelet levels around a week of exposure to the new drugs, and this may lead to a risk of bleeding. This occurs at an estimated rate of 10 cases per million per annum, and the mortality is almost 1.5％-6.2％ in a few recent studies of epidemiology. The commonly implicated drugs are quinine, penicillin, sulfamethoxazole, etc. The important thing to consider in drug-induced thrombocytopaenia is to exclude other potential causes linked to the problem. However, patients need frequent monitoring due to the risk of fatal bleeding [1-3]. 

This antibiotic is quite effective due to its actions against a wide variety of gram-positive bacteria. It is administered intravenously and is based on body weight. Additionally, this is structurally related to ristocetin, which is known to lead to thrombocytopaenia due to agglutination effects [4]. This drug-induced thrombocytopaenia may sometimes be challenging to diagnose, as a number of causes might be the culprits in hospitalised patients [5,6].

It is recommended that full blood count be monitored while someone is on this drug for a week or more because it may cause hematological side effects by various mechanisms. One of the common side effects is thrombocytopaenia, which is usually seen after drug exposure for a week or more. However, it reverses once the drug is stopped. However, other causes of low platelet count should be excluded at the same time, and the patient’s safety should be ensured with frequent monitoring for small or fatal bleeding due to low platelet count.

## Case presentation

This case presentation is about an 83-year-old man who was admitted with feeling unwell and increased pain and swelling of his legs (Right >Left). On examination, all other systems were within normal limits, but the legs were erythematous and swollen, especially the right leg. Blood tests showed raised infection markers, i.e., white cell count, and he was started on empiric antibiotics for cellulitis of the right leg. Blood cultures and wound swabs were sent. Blood cultures were negative, but wound culture grew Staphylococcus aureus. This was sensitive to teicoplanin and doxycycline. Having markedly raised infection markers, he was treated with teicoplanin. His initial full blood count is shown in Table 1 as Day 1 Results.

**Table 1:** Different components of a full blood count panel at different stages of treatment and one week post treatment. Hb, hemoglobin; MCV, mean corpuscular volume; HCT, hematocrit; MCH, mean corpuscular hemoglobin; MCHC, mean corpuscular hemoglobin concentration; RDW, red cell distribution width.

Blood Parameters	Reference Range	Day 1 Results	Day 11 Results	Results One-Week Post-treatment
Hb (g/L)	130 - 180	128	117	116
WBC (x10^9^/L)	4.0 - 13.0	14.5	7.2	9.3
Platelets (x10^9^/L)	135 - 400	291	78	220
MCV (fL)	80 - 98	91	88	87
HCT (l/l)	0.4- 0.5	0.376	0.376	0.345
RBC (x1012/L)	0-0	4.1	3.7	4.0
MCH (pg)	27-35	31	31	31
Neutrophils (x10^9^/L)	2.0 – 7.5	12	6.5	7.2
Lymphocytes (x10^9^/L)	1.0 – 4.0	1.1	1.1	1.1
Monocytes (x10^9^/L)	0.2 - 0.8	0.80	0.22	0.64
Eosinophils (x10^9^/L	0.04 - 0.4	0.1	0.1	0.1
Basophils (x10^9^/L)	0.0 – 0.1	0.03	0.03	0.03
Nucleated red cells (x10^9^/L)	0-0	0.0	0.0	0.0

At day 7, the teicoplanin level was checked, and it was in the therapeutic range. Full blood count was also repeated every 2-3 days, and it showed improvement in white cell counts. However, at day 10, this patient’s platelet count was 78,000/microL. This is shown in Table 1 Day 11 Results. 

The next day, it dropped to a further lower level, and it was 18,000 x 10^9^/L. This patient was on Apixaban 5mg BID for atrial fibrillation. This was held for now and a search for the causes of low platelets was started. After checking for the possible causes, it was found that teicoplanin is most likely the offending agent. It was stopped on the same day, and platelets were monitored further. This patient was observed more frequently due to the possibility of spontaneous bleeding. He developed petechiae all over his body that were monitored and were due to thrombocytopenia. However, over the next few days, he improved clinically as the petechiae vanished after improvement in the platelet count. His platelet count improved gradually to 30,000 x 109/L, then 44 x 10^9^/L, and reached normal levels of 220 x 10^9^/L within a week after stopping teicoplanin. This is shown in Table 1 Results 1-week post treatment.

Other causes of thrombocytopenia i.e., disseminated intravascular coagulation, nutritional deficiencies, and heparin-induced thrombocytopaenia, etc., were excluded.

## Discussion

Thrombocytopaenia is defined as a low platelet count in the blood. There is a broad differential in what can cause thrombocytopaenia. In the context of the case, it would be classified as drug-induced thrombocytopaenia. The pathogenesis of this entity can be classified into decreased platelet production, such as in bone marrow suppression (e.g., cytotoxic drugs used in chemotherapy), or accelerated platelet destruction, which can be further classified into non-immune-mediated and immune-mediated. 

This drug-induced thrombocytopaenia has various causes, of which there are six distinct aetiologies: immune complex mediated, as seen with heparin, which is one of the most common forms, drug-specific antibody, as seen with abciximab, autoantibody induced as seen with gold salts and procainamide usage, fibrin induced with drugs such as eptifibatide, hapten-induced antibody, as seen with penicillin, and drug-dependent seen in quinine use. 

It has been proposed that drug-induced thrombocytopaenia is caused by drug-dependent antibodies, which bind to specific epitopes on platelet surface glycoproteins in the presence of a specific drug. These drug-dependent antibodies are specific to the drug structure. It is proposed that these sensitizing drugs bind noncovalently to platelets, most commonly GP IIb-IIIa and/or GP Ib-V-IX site, and occasionally platelet endothelial cell adhesion molecule, and are reversible in nature. A model has been made which highlights this and proposes the reversible binding of drugs and platelets, denoting a ‘sandwich’ like mechanism whereby a tight bond between platelet and antibodies is formed. This immune-mediated reaction usually occurs one to two weeks after being exposed to said drug, and as the literature states, platelets are more often the target for drug-dependent antibodies than other cells [7,8].

Drug-induced thrombocytopaenia can also be assessed by the use of the WHO causality scale, which includes the time of event, offending agent, and exclusion of any other competing cause, response after drug withdrawal or dose reduction, which is called de-challenge. Furthermore, response to the re-administration of the same agent and see the response, this is called re-challenge [9].

This drug-induced thrombocytopenia is an uncommon finding epidemiologically and is seen in patients receiving any agent that may potentially affect platelets. The common causative drugs are chemotherapeutic drugs, penicillin, furosemide, and gold, used to treat inflammatory conditions [10].

The clinical features are the same as any thrombocytopaenia, such as petechiae, purpura or bleeding, i.e., haematuria, epistaxis. However, the causes of this thrombocytopaenia are numerous, and differential diagnoses include Immune thrombocytopaenia, Heparin-induced thrombocytopaenia, sepsis, and nutritional deficiencies. Furthermore, the management is carefully monitoring, investigating for causes, and treating the cause, if any [11].

Teicoplanin is a glycopeptide-class antibiotic used to treat gram-positive infections. A study done in 2005 took a cohort of 39 patients being treated with teicoplanin. Thirty-one of these patients already had thrombocytopaenia (platelets 1-105 × 10⁹/L) and eight had normal platelet levels. The study employed flow cytometry, IgG detection assays and ELISA. The results indicated 45% (14 subjects) of the thrombocytopenic cohort had detectable IgG antibodies that bind to platelets in the presence of teicoplanin. It also showed that out of the cohort of subjects that had a normal thrombocyte count 25% (2 subjects) developed antibodies specific to teicoplanin without showing any drop in platelet count. This denotes that even if antibodies are formed whilst being treated with teicoplanin, it doesn’t necessarily mean that thrombocytopaenia will develop [12]. The study highlights a drug-dependent immune mechanism whereby thrombocytopaenia is caused by teicoplanin binding to the platelets, exposing the epitopes of the GP IIb-IIIa, which are recognized by IgG antibodies, concluding that the primary target for the antibodies is the platelet glycoprotein GP IIb-IIIa complex. 

## Conclusions

In conclusion, the patient being treated on teicoplanin for a week or more should be monitored for thrombocytopaenia due to a potential drop in platelets. This is due largely to the mechanism of platelet aggregation. This case report of a patient who needed antibiotics for infection and then ended up with critically low levels of platelets highlights the importance of timely monitoring with a full blood count in order to prevent any further complications. This is a rare case, but an important aspect is to recognise it earlier. 
